# Predictors of Student Productivity in Biomedical Graduate School Applications

**DOI:** 10.1371/journal.pone.0169121

**Published:** 2017-01-11

**Authors:** Joshua D. Hall, Anna B. O’Connell, Jeanette G. Cook

**Affiliations:** 1 Office of Graduate Education, University of North Carolina School of Medicine, Chapel Hill, NC, United States of America; 2 Department of Biochemistry and Biophysics, University of North Carolina School of Medicine, Chapel Hill, NC, United States of America; Charles University, CZECH REPUBLIC

## Abstract

Many US biomedical PhD programs receive more applications for admissions than they can accept each year, necessitating a selective admissions process. Typical selection criteria include standardized test scores, undergraduate grade point average, letters of recommendation, a resume and/or personal statement highlighting relevant research or professional experience, and feedback from interviews with training faculty. Admissions decisions are often founded on assumptions that these application components correlate with research success in graduate school, but these assumptions have not been rigorously tested. We sought to determine if any application components were predictive of student productivity measured by first-author student publications and time to degree completion. We collected productivity metrics for graduate students who entered the umbrella first-year biomedical PhD program at the University of North Carolina at Chapel Hill from 2008–2010 and analyzed components of their admissions applications. We found no correlations of test scores, grades, amount of previous research experience, or faculty interview ratings with high or low productivity among those applicants who were admitted and chose to matriculate at UNC. In contrast, ratings from recommendation letter writers were significantly stronger for students who published multiple first-author papers in graduate school than for those who published no first-author papers during the same timeframe. We conclude that the most commonly used standardized test (the general GRE) is a particularly ineffective predictive tool, but that qualitative assessments by previous mentors are more likely to identify students who will succeed in biomedical graduate research. Based on these results, we conclude that admissions committees should avoid over-reliance on any single component of the application and de-emphasize metrics that are minimally predictive of student productivity. We recommend continual tracking of desired training outcomes combined with retrospective analysis of admissions practices to guide both application requirements and holistic application review.

## Introduction

The PhD degree is required for advancement to leadership within biomedical research fields. As a consequence, graduate school admissions acts as a *de facto* filter for scientific leadership opportunity. PhD programs often receive many more applications from qualified candidates than the number of training slots available, leading to intense competition during the admissions process [1]. For example, the University of North Carolina at Chapel Hill typically receives 1,100–1,300 applications to the umbrella biomedical PhD program each year, but the typical matriculating class is only 80–90 students. To select candidates from a large pool of qualified applicants, committees must look for aspects of the application that differentiate candidates in a meaningful way. With limited information in the graduate application, this can be a difficult process and can lead committees to overly rely on quantitative metrics like standardized test scores or grade point averages for quick comparisons [2]. Despite the importance of the application process, applicant characteristics presumed to predict success in the biomedical sciences are based largely on untested assumptions. Moreover, it is critical to rigorously examine selection criteria to reduce or eliminate factors that introduce biases that disproportionately limit certain groups’ access to PhD training and the biomedical workforce.

Biomedical PhD programs provide highly varied training experiences involving specialized technical training, critical analysis of data and the scientific literature, problem solving, and both informal and professional communication. Selection criteria for biomedical graduate programs have been relatively consistent over the past decades and include Graduate Record Examination (GRE) scores, undergraduate grade point average (GPA), letters of recommendation, and a resume and/or personal statement highlighting past research and professional experiences. While scientific PhD programs train students to think critically and rigorously test hypotheses, the effectiveness of the graduate school admissions process itself has not been fully examined. A recent analysis of biomedical graduate students at the University of California San Francisco (UCSF) found that standard application metrics such as general GRE test scores, grades, and undergraduate institution ranking were not predictive of graduate student success [3]. However, some UCSF graduates disputed the subjective nature of “success” that was utilized as an outcome variable in the original study [4]. To build upon the findings of Weiner with a larger and independent cohort, we tested graduate school application components for correlations with objective measures of productivity–namely, publications and time-to-degree.

The goal of this study was to examine factors considered by admissions committees at the University of North Carolina at Chapel Hill (UNC) when assessing applicants for the Biological and Biomedical Sciences Program (BBSP), an umbrella admissions program comprised of 14 PhD programs in the UNC Schools of Medicine, Pharmacy, and Dentistry, and the College of Arts and Sciences at UNC Chapel Hill. Each year, BBSP admits approximately 85 students who matriculate into one of the 14 life sciences PhD programs at the end of the first academic year. This centralized biomedical PhD admissions and training program was formed in 2008, and the scale of the admissions activity from this combined academic effort facilitated the analysis of application and admissions data for students who were admitted from 2008–2010. Our goal was to assess the research productivity of these students, as measured by publications and time-to-degree, and to identify which admissions factors, if any, were predictive of their performance. Among the metrics assessed, we found that only recommender evaluations could distinguish between the most and least-productive graduate students. These results have implications for refining the graduate student selection process to simultaneously reduce bias and select students with a higher likelihood of desired outcomes.

## Methods

The cohort studied comprised 280 graduate students who entered the BBSP at UNC from 2008–2010; 195 had graduated with a PhD at the time of this study (July 2016), 45 were still enrolled, and 40 graduated with a Master’s degree or withdrew. The cohort included all of the BBSP students who matriculated from 2008–2010. All application metrics (GRE scores, undergraduate GPA, letters of recommendation, and previous research experience) were recorded from each student’s BBSP application. Interview scores were calculated as an average of one-on-one student interviews with (typically) five BBSP-affiliated faculty members. This study was an analysis of publicly available publication data and existing student application data. Data collection was reviewed by the Office of Human Research Ethics at UNC Chapel Hill, which determined that this submission (study #140544) does not constitute human subjects research as defined under federal regulations [45 CFR 46.102 (d or f) and 21 CFR 56.102(c)(e)(l)] and does not require IRB approval.

### GRE Scores and GPA

The GRE is a timed, standardized examination administered by the Educational Testing Service in the US and other countries. The test is divided into three parts: Quantitative reasoning (math) and Verbal reasoning, and Writing, which involves writing two time-limited essays. GRE scores (Quantitative, Verbal, and Writing) were taken from each student’s BBSP application. If a student took the GRE multiple times, the highest reported score for each subsection was used for admissions decisions and for this study. GRE percentile scores were used in our analysis. Grade point average (GPA) is an average of a student’s performance in coursework during their academic studies. Each student’s most recent undergraduate (i.e. college) GPA was also taken from their BBSP application and used for this analysis.

### Previous Research Experience

Months of previous research experience were manually calculated from each application based on information found in the applicant’s CV, personal statement, and letters of recommendation. Part-time research experience was converted to full-time months by multiplying the number of part-time months by 0.375. This conversion is based on NIH guidelines for tabulating research experience for T32 training grant tables (https://www.nigms.nih.gov/training/Pages/New-Training-Tables-FAQs.aspx). Months of previous research experience were calculated only up to the date of the application (December of the year prior to entry into graduate school), and does not include likely additional research in the spring and summer prior to matriculation. Participation in laboratory components of science courses was not counted as research experience.

### Recommendation Letter Writer Ratings

Each BBSP application included three letters of recommendation, typically from previous research advisors. In addition, letter writers rated the applicant as “Exceptional”, “Outstanding”, “Very Good”, “Above Average”, or “Below Average”. These ratings were converted to a numerical score where Exceptional = 1 and Below Average = 5. In some cases, one or more letters were missing or the recommender rating was missing. Only students with three recommender ratings were included in the analysis of recommender ratings (n = 251).

### Interview Scores

A subset of BBSP applicants was selected to visit the UNC campus. The itineraries for these visits included five 30-minute, one-on-one interviews with BBSP faculty who submitted feedback about the applicants for consideration by the admissions committees when deciding which candidates would receive offers of admission. As part of the feedback, faculty recommended students for admission on a 5-point scale where 1 = “recommend highly”, and 5 = “do not recommend”. Records of these ratings were only available for the 2009 and 2010 cohort. Only students with at least 4 faculty interview scores were included in our analysis (n = 142).

### Student publications

Publications by each student during graduate school were quantified with a custom Python script that queried Pubmed (http://www.ncbi.nlm.nih.gov/pubmed) using author searches for each student’s name paired with their research advisor’s name. The script returned XML attributes (https://www.nlm.nih.gov/bsd/licensee/elements_alphabetical.html) for all publications and generated the number of first-author publications and the total number of publications (including middle authorship) for each student/advisor pair. This script is available upon request. All student/advisor combinations returning no publications were checked manually to ensure there were no special circumstances that would interfere with the query (for example, student name change, advisor change, etc). A random subset of student publication data was also checked manually. All publications up to July 12, 2016 were included in this analysis.

### Student Outcomes and Statistical Analysis

Students were grouped into four bins based on their number of publications during graduate school: “3+” = students with ≥ 3 first-author publications; “1–2” = students with 1 or 2 first-author publications; “0+” = students with no first-author publications, but at least one middle author publication; and “0” = students with no publications. All publications were counted equally including primary research papers, review articles, highlights, perspectives, etc. Due to the non-parametric distribution of these metrics within our cohort, application metrics were compared among these groups of students by a Kruskal-Wallis test, and a p-value of < 0.05 was considered to be significantly different. In situations where the Kruskal-Wallis test returned a p<0.05, direct comparisons between specific groups were made using Dunn’s multiple comparisons test, and a p-value of < 0.05 was classified as a significant difference. It is worth noting that assessing differences using ANOVA and Tukey’s multiple comparisons test yielded identical conclusions as Kruskal-Wallis and Dunn’s test, likely due to the relatively large size of our study cohort.

## Results

To test for correlations between application components and graduate student productivity, we collected applications for admissions and publication data for the cohort of 280 students who matriculated into the UNC umbrella first-year program, BBSP, between 2008 and 2010. Descriptive information about the study cohort is included in Table 1. The demographics of this cohort were 61.4% female and 22.9% from racial/ethnic groups that are underrepresented in the sciences (African American, Hispanic/Latina/o, Native American, Hawaiian or Pacific Islander). The program is selective and receives approximately 1,300 applications each year. The admissions committees narrow the pool to approximately 300 applicants for on-campus interviews, and from that group, 220–250 are offered admission in a typical application year. An average of 85 students matriculate into the BBSP each fall. At the time of this study, >85% of the 2008–2010 BBSP students had either graduated with a PhD or were still making progress towards graduation; the average time to degree was 5.5 years.

**Table 1:** Study population descriptive statistics.

	N			N
**Gender**			**Enrollment Status**	
Female	172		Still Enrolled^a^	45
Male	108		Graduated PhD	195
			Graduated MS	14
**Race/Ethnicity**			Withdrew	26
Asian	30			
Black/African American	36		**Publication Groups**	
Hawaiian/Pacific Islander	4		3+	50
Hispanic/Latina/o	20		1–2	151
Native American	4		0+	41
White	179		0	38
Other/Unsure	7			
			**Overall Means**	
**Starting year**			Quantitative GRE Percentile	72.48+/-17.47
2008	123		Verbal GRE Percentile	73.10+/-19.30
2009	84		Writing GRE Percentile	54.28+/-22.15
2010	73		Undergraduate GPA	3.52+/-0.34
			Previous Research Experience (months)	18.33+/-16.75
			Recommendation Letter Rating^b^	1.74+/-0.45
			One-on-one Interview Score^c^	1.90+/-0.38
**TOTAL**	280		First-Author Publications	1.45+/-1.40

Individuals included in this study were PhD students who entered the Biological and Biomedical Sciences Program (BBSP) from 2008–2010. Students were assigned to the following Publication Groups based on number of first-author publications during their graduate studies: 3+, ≥3 first-author publications; 1–2, 1 or 2 first-author publications; 0+, 0 first-author publications and at least one middle authorship; and 0, no first-author or middle-author publications.

^a^ Students still enrolled and making progress towards degree at the time of submission.

^b^ Only includes students with at least 3 recommendation letter ratings (n = 251)

^c^ Data only available for students from the 2009–10 cohorts; only includes students with at least 4 faculty interview scores (n = 142)

To assess graduate student productivity, we quantified the number of first-author publications, a measure of independent work that is often utilized as a PhD completion requirement. Since all of the 14 BBSP-participating programs require 1–2 first-author publications for PhD completion, we used publications as a proxy for graduate student productivity. It is worth noting that BBSP students are typically successful with most (72%) having at least one first-author publication at the time of this study. We sought to determine if the most productive graduate students, i.e. those with the most first-author publications, had quantifiable differences in their graduate school applications compared to graduate students that had fewer or no publications. In addition, we compared application data among students with varying time to degree and PhD completion status.

We grouped students into four bins based on the number of publications associated with their graduate studies as defined by co-authorship with their primary thesis advisor at any point during or after graduation. We defined highly productive students as those with 3 or more first-author publications and students with 1 or 2 first-author publications as having shown average productivity (1 or 2 first-author publications is the typical graduation requirement for biological and biomedical departments at UNC). We counted all publications irrespective of type (review or primary data report) or journal. For those students with no first-author publications, we subdivided them into those with at least one middle authorship and those with no publications of any kind from their work as a graduate student. We designated these groups “3+”, “1–2”, “0+”, and “0”, respectively. We were most interested in distinguishing between students who met the research expectations for the PhD–at least one first-author paper–and those who did not. We chose to further subdivide those two groups at the outset of the analysis to also identify those who were highly productive (more than 3 first author papers) and those that were very minimally productive (no papers at all).

**Fig 1:**
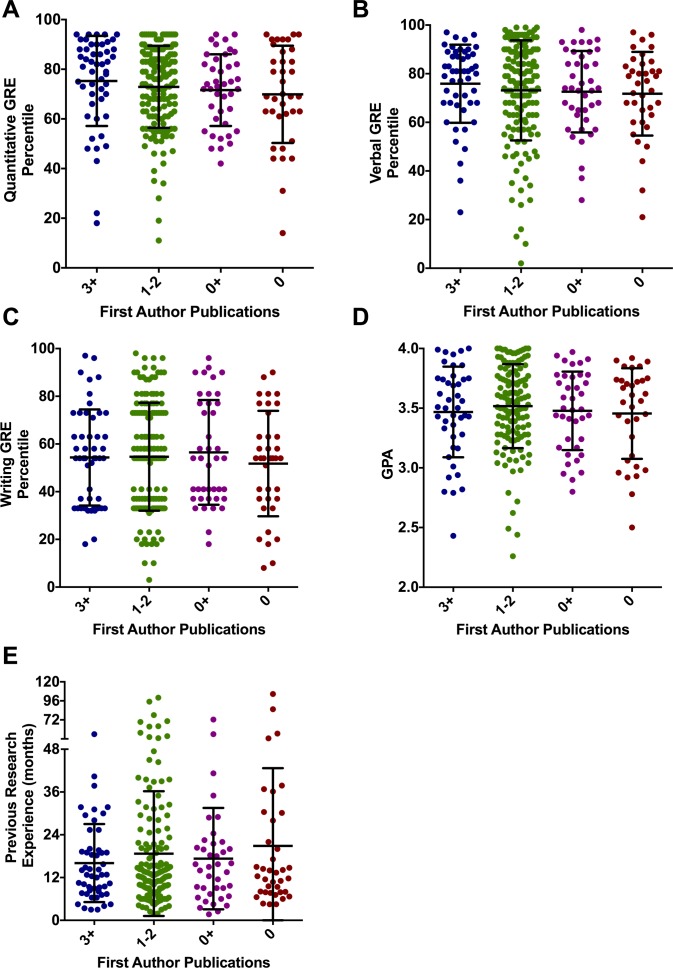
Graduate student application metrics vs. publication productivity. Students from the 2008–2010 entering classes were assigned to the following groups based on number of first-author publications during their graduate studies: 3+, ≥3 first-author publications; 1–2, 1 or 2 first-author publications; 0+, 0 first-author publications and at least one middle authorship; and 0, no first or middle-author publications. (A) Quantitative GRE scores, (B) Verbal GRE scores, (C) Writing GRE scores, (D) Undergraduate GPA, and (E) previous research experience were compared among the groups of students. Each symbol represents one student, and lines and error bars represent the mean and standard deviation of each population, respectively. A Kruskal-Wallis test was used to assess differences among the populations, and p-values for comparisons among the groups in panels A, B, C, D, and E were 0.3251, 0.6165, 0.8460, 0.7625, and 0.9896, respectively.

The BBSP application includes academic transcripts, general GRE scores (quantitative, verbal, and writing but not a subject test), a personal statement, a CV/resume of past academic and vocational experiences, and three letters of recommendation. To determine if GRE or GPA were predictive of biomedical graduate student productivity, we compared mean GRE percentile scores and undergraduate GPA among students with varying numbers of publications from their graduate study. There was no statistical difference among these groups with regard to quantitative GRE score, verbal GRE score, writing GRE score, or GPA (Fig 1A–1D). On the other hand, we found that the quantitative GRE scores in our cohort differed by gender and race/ethnicity; males scored higher than females and Asian and white test takers scored higher than those from under-represented minority groups (data not shown), similar to observations for all science graduate school test takers reported by Miller and Stassun [5]. Notably, a substantial number of students with below-average GRE scores were ultimately quite productive whereas some students with near-perfect GRE scores were minimally productive in graduate school. These findings parallel those of Weiner (2014) and most recently, Moneta-Koehler et al. [6], and they reinforce doubts about the usefulness of GRE scores in admissions for biomedical PhD programs.

Given that a prior study suggested that research experience correlated with graduate student success (as determined qualitatively by graduate program leadership) [3], we compared the amount of previous research experience among UNC graduate students for each productivity group. We only counted months of research experience reported by students in the application. We converted part-time months to full-time months as outlined in Methods, thus the values reported here are not strictly the total length of time that applicants were associated with a research group. It is also important to note that the vast majority of applicants likely remained actively engaged in research after the December submission date, which would add an additional 5–7 months of research prior to matriculation in August; however, we could only accurately quantify research experience listed in the application submitted in December prior to the year of matriculation. For this reason, our research experience metrics in Table 1 and Fig 1E are almost certainly an underestimate, though likely uniformly underestimated across groups. Surprisingly, there was no difference in the amount of previous research experience among our most and least productive students (Fig 1E).

**Fig 2:**
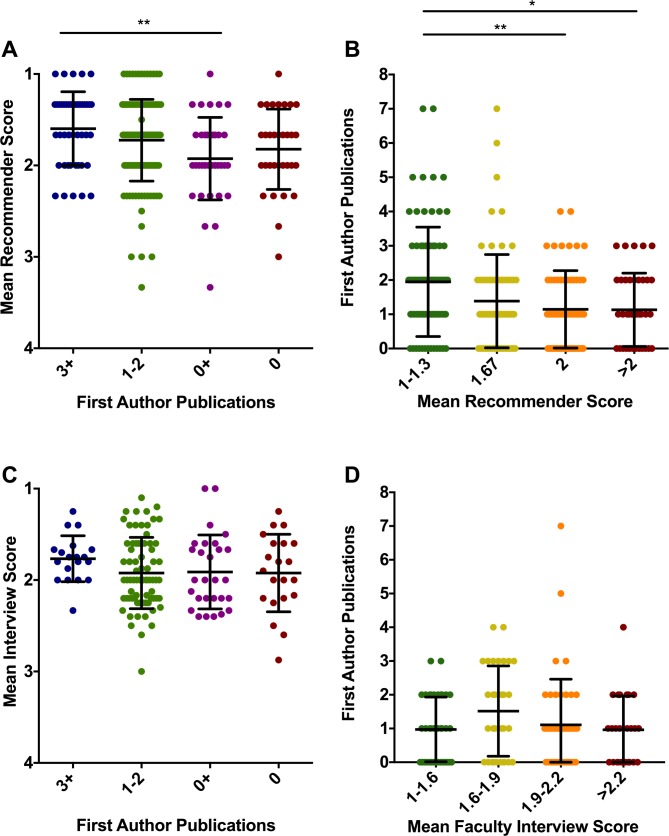
Recommender evaluations predict graduate student publication productivity. Students were assigned to groups according to first-author publications as in Fig 1. (A) Ratings from recommendation letters associated with their graduate school applications were converted from the adjective selected by the recommender (from the UNC-provided options of “Exceptional”, “Outstanding”, “Very Good”, “Average”, or “Below Average”) to a numerical score (1 = Exceptional, 5 = Below Average), averaged, and compared among the groups of students. (B) To assess whether students with the highest recommender ratings were more productive, students were assigned to groups according to their mean recommender score from the three letters, and the number of first-author publications was plotted for each group. (C) As in A except that the mean score from one-on-one faculty interviews was plotted (1 = most enthusiastic, 4 = least enthusiastic). (D) To assess whether students with the highest interview scores were more productive, students were binned according to their mean one-on-one faculty interview score, and the number of first-author publications was plotted. Each symbol represents one student, and lines represent the mean and standard deviation of each population A Kruskal-Wallis test was used to assess differences among the populations, and p-values for comparisons among the groups in panels A, B, C, and D were 0.0060, 0.0050, 0.3459, and 0.3072, respectively. For comparison between specific groups, Dunn’s multiple comparisons test was performed (* p < .05, ** p < .01).

Letters of recommendation are a valuable component of the graduate application because they provide a detailed and expert assessment of relevant ability by individuals who have observed the student’s work over time. We therefore hypothesized that the ratings of letter writers might predict graduate student productivity. In addition to writing a letter of reference, recommenders provided an overall rating of the applicants as “Exceptional”, “Outstanding”, “Very Good”, “Average”, or “Below Average”, which we then converted to 1, 2, 3, 4, or 5, respectively. Using these metrics, we calculated a mean recommender score for each application. Remarkably, students with 3+ first-author publications had higher mean recommendation letter ratings (1.60+/-0.40) than those in the 0+ (1.93+/-0.45) or 0 (1.82+/-0.44) groups, though only the difference between the 3+ and 0+ group met our stringent significance criteria when multiple comparisons were accounted for (Fig 2A, 3+ vs 0+, p = 0.0052; 3+ vs 0, p = 0.1761, Dunn’s multiple comparisons test).

To further assess the ability of recommender ratings to predict student productivity, we tested whether students with the most enthusiastic recommender ratings had greater research productivity than students with less enthusiastic recommender ratings. For this test, we assigned applicants to groups based on their mean recommender score. The best theoretical recommender average was a 1 (three letter ratings of “Exceptional”). Students in the strongest group had recommender scores of 1 or 1.33 (n = 78), followed by students with average recommender scores of 1.67 (n = 73), 2 (n = 62), and >2 (n = 38). Students with the best average recommender ratings in their graduate school application authored significantly more first-author publications during graduate school (1.92+/-1.60) than either of the two groups with weaker average recommender scores (1.15+/-1.13 publications for the group with an average recommender score of 2 and 1.13+/-1.07 publications for the group with recommender scores greater than 2.) (Fig 2B, p = 0.0968, 0.0098, and 0.0465, for “1–1.3” group vs “1.67”, “2”, and “>2” groups, respectively, via Dunn’s multiple comparisons test). Thus, letter writer ratings were predictive in determining which students ultimately produced multiple first-author papers.

Like many biomedical graduate programs, UNC BBSP utilizes a two-part review process. After applications were read and scored by admissions committees in the first round, a subset of applicants were invited to UNC for further evaluation in a series of 30 minute, one-on-one interviews with faculty. The interviewers provided feedback about the applicants, which gave admissions committees additional insight beyond what could be gleaned from the written application. Interviewers submitted an online survey after each meeting and, in addition to comments about the applicant, selected from one of 4 scores with the following descriptors: 1 - “Accept without reservation”, 2 –“Accept”, 3 –“Accept if space available”, and 4 –“Reject”. In contrast to ratings by recommendation letter writers, one-on-one interview scores did not distinguish the most productive graduate students from the least productive (Fig 2C). Likewise, the students with the highest average interview scores did not publish more papers than students with lower interview scores (Fig 2D).

Finally, we separated our cohort into five groups based on time-to-degree and degree completion status (completed PhD in <5 years, completed PhD in 5–6 years, completed PhD in >5 years, exited prematurely with a Master’s degree, or withdrew). There were no statistical differences among these groups with respect to GRE scores (S1A–S1C Fig), GPA (S1D Fig), previous research experience (S1E Fig), mean letter writer score (S1F Fig), or one-on-one interview score (S1G Fig). Overall, we conclude that the assessment of other scientists who have observed the applicant in a research setting is the most predictive of the number of student research publications resulting from their biomedical PhD training.

## Discussion

A primary goal of biomedical PhD training is developing high-level scientific skills through the process of generating a significant body of original published research. A previous study [3] utilized subjective faculty assessments to group students into “high” and “low” performers, which prompted criticism from a subset of the student cohort as potentially biased [4]; this consideration influenced our desire to use an impartial measure of productivity. We thus chose to test for correlations between application components and first-author papers, though we acknowledge that the simple counting of publications is an imperfect measure of productivity. Publication frequencies vary among disciplines and laboratories, and certainly one highly influential paper may represent a substantial body of work, whereas three or more small papers may be considered only average productivity in some cases. Furthermore, we chose not to use impact factors or other journal metrics, in part because of the many disciplines that comprise the BBSP, and also because of the various weaknesses and potential biases associated with such metrics [7]. Despite the limitations, we assert that publications provide an objective, broad, and useful dataset to assess retrospectively the admissions process that initially selected UNC biomedical PhD students.

We chose to bin students into four groups as a useful framework for comparing student research productivity, especially when comparing graduate students with 3+ first author publications to their peers with no first-author publications at all in the same time frame. In addition, 1–2 first author papers is a graduation requirement for UNC BBSP-affiliated programs, and thus, this threshold provides a practical comparison point for grouping students who were more and less productive than this typical standard. We further note that “student success” is multifaceted, and students with few papers may be very well prepared for a variety of careers, including in academia. Indeed, “success” from the students’ perspective is likely more complex than from their thesis advisors’ or institutional leadership’s perspective [4, 8]. For this reason, we were careful to limit our description of this study to student research productivity and not student “success”.

In contrast to widely held assumptions by admissions faculty and administrators about the predictive power of grades and general GRE test scores, we found no correlation between these metrics and student publications or degree completion. We note that although undergraduate grades in our cohort were typically strong (mean GPA 3.52+/-0.34), the range of general GRE scores included both very high and relatively low test performances. The lack of correlation of GRE scores and PhD student productivity is consistent with the analysis of UCSF students [3], as well as a more recent study of the biomedical graduate student population at Vanderbilt University Medical School [6]. It is not surprising, in retrospect, that classroom performance or success in a time-limited testing environment does not correlate with long-term research achievement since these activities differ substantially. Although ETS, the company that administers the GRE, markets to institutions that, “GRE scores are a proven measure of an applicant's readiness for graduate-level work—and of their potential for success” [9], evidence of the GRE’s ability to predict success in graduate school is scant, and the strength of correlations between test scores and student outcomes may vary widely among academic disciplines. Perhaps in response to recent critiques about the utility of the test, ETS recently issued a statement that discouraged graduate schools from heavy reliance on GRE scores for admissions decisions [10].

Miller and Stassun (2014) recently analyzed ETS data from US test takers and found clear differences in quantitative reasoning GRE scores among candidates in STEM disciplines from different ethnic groups and genders. Our analysis found these same correlations in our cohort. For this reason, we conclude that reliance on the GRE introduces unintended bias against certain groups that historically perform less well on the exam without actually increasing excellence in the matriculating class. Graduate admissions committees that base admissions decisions on GRE performance may be relying on measures that are minimally predictive of desirable student outcomes, but maximally predictive of race and gender. This outcome is of particular concern since building creative and innovative research teams is enhanced by diverse individual perspectives [11–13], and building such teams requires increased participation by under-represented groups. Our findings also match Weiner (2014) with respect to GPA, indicating that some of the most common quantitative measures for graduate school admission are incapable of reliably predicting which applicants will be productive graduate students.

We were somewhat surprised that higher amounts of prior research experience did not correlate with productivity, a finding in contrast to Weiner (2014), which was a principal inspiration for our analysis. One potential explanation is that our study included considerably more students than the UCSF study (280 vs 52). It should also be emphasized that virtually all students accepted into the UNC program had substantial previous research experience (note that our metric converts part-time months to full-time months and underestimates total research prior to matriculation); thus, we had no control population of accepted students with little-to-no previous research experience. Nonetheless, our cohort had a wide range in months of prior research experience, and our data suggest that those with substantially more research experience did not necessarily fare better in graduate school.

We were also surprised that the (subjective) UNC faculty scores from one-on-one interviews did not distinguish the most productive from the least productive students. It is possible that 30 minutes of conversation in an interview setting is not enough interaction to distinguish future high-level productivity within a cohort of generally highly qualified applicants. On the other hand, these meetings were likely effective in identifying applicants who were an excellent scientific match for UNC labs and importantly, may have identified those who were not a good match for UNC and thus were not suitable for an admissions offer (and thus not included in our analysis).

We are cognizant of several limitations of our study, a principle one being that we could only analyze applications and outcomes for students who were admitted to UNC and chose to attend. For example, we do not know if those candidates we declined to offer admissions (either on the basis of their submitted application or the one-on-one interview) were in fact less productive. Moreover, we clearly could not compare UNC applications and outcomes to cohorts at other institutions. Nonetheless, we are encouraged by the general concordance of our study with those at UCSF and Vanderbilt [3, 6]. A further limitation was that we examined application components in isolation. We emphasize that our conclusions may only apply to admissions outcomes in the experimental biological and biomedical sciences at institutions with similar admissions practices, and that other disciplines may find substantially different correlations. Our data suggest that the most useful application review approach will be a holistic and multivariate one that gives appropriate relative weight to each part of the application.

Despite these limitations, a striking and potentially useful finding was that recommender scores can predict student productivity. This observation highlights the value of feedback from individuals who have observed the student over time in a research setting. We note that most letters for accepted UNC students are positive, which is not surprising since a faculty member is unlikely to agree to provide a letter for an applicant they do not consider qualified for graduate work. We are thus intrigued by the range in selected ratings when recommenders were given a choice among several positive adjectives of varying strength. We speculate that a letter writer will produce a generally positive letter for an above-average applicant, but will only select “exceptional” (or a homologous top rating) for those candidates who have shown the constellation of characteristics that typically correlate with research success. Those who excel in scientific pursuits may be those who persevere and maintain focus and optimism in the face of regular challenges, and these traits may inspire enthusiastic ratings from referees. In support of the importance of such traits, a study of 100 dental students found multiple emotional intelligence competencies that differentiated average from outstanding students with regard to their performance in assessments that mimicked professional activity [14]. These competencies, which included emotional self-control, achievement orientation, initiative, trustworthiness, conscientiousness, adaptability, and optimism were predictors of mean clinical grades assigned by preceptors. In contrast, didactic measures such as Dental Admission Test (DAT) scores and GPA were not predictive. A better understanding of traits that contribute to student researcher productivity may inform training institutions about skill areas for intentional development [15, 16].

While our results provide evidence that feedback from recommenders does have some predictive value for selecting productive graduate students, our study does not support the conclusion that faculty should rely solely on letters of recommendation when evaluating graduate applications. First, like other evaluation metrics, recommendation letters may contain biases [17]. In that regard, biases in the letter texts themselves have not been measured or taken into account here, though analysis of the texts of both the letters and personal statements is a future goal. In an attempt to mitigate the effects of such biases in our own BBSP admissions process at UNC School of Medicine, we now include discussion of unconscious bias research with admissions committee faculty prior to the application review process.

A final, but important, consideration when evaluating student productivity is that studies such as this (and others) tend to focus primarily on the contribution of *student* characteristics to graduate training outcomes [3, 6]. We assert that a great many factors contribute to overall student productivity that cannot be measured in an application review or interview and are both external to the student and/or develop after matriculation. Importantly, scientific training involves a close relationship between student and research advisor. The funding level, management style, and overall productivity of the principal investigator likely has a substantial influence on graduate student productivity. We acknowledge that factors contributing to productivity and ultimate success in scientific PhD programs are complex and include the attention and resources of the mentor, training provided by the institution, the scientific match of the student to the discipline, the availability of collaborators, social support networks, and serendipity. Nonetheless, admissions decisions must be made with necessarily limited information, and we anticipate that continued analysis, such as we have begun here and similar studies at peer institutions, will improve the objectivity and effectiveness of the admissions process.

In conclusion, we provide evidence that traditional quantitative metrics used in graduate school admissions are minimally predictive of future student productivity. In particular, our findings argue against over-reliance on single metrics (such as the GRE) that are minimally predictive of desired outcomes, but disproportionately bias against certain groups. In contrast, our findings indicate that qualitative assessments in recommendation letters are valuable in predicting which students will be most or least productive in biomedical PhD programs. We also suggest that examining and adjusting application requirements and interview practices to focus admissions decisions on the most relevant traits for success in a given discipline will improve the graduate admissions process. Holistic assessment of graduate applicants based on broad evidence-based understanding of the relative strengths of application components will ensure opportunities for the most promising candidates.

## Supporting Information

S1 Fig Standard graduate school metrics do not predict PhD student completion or time-to-degree. Students were assigned to groups based on their graduate school outcome, and students who completed their PhD were grouped based on time-to-degree (< 5 years, 4–5 years, > 5 years). (A) Quantitative GRE scores, (B) verbal GRE scores, (C) writing GRE scores, (D) GPA, (E) previous research experience, (F) recommender scores (1 = highest, 4 = lowest), and (G) one-on-one interview scores (1 = highest, 4 = lowest), were compared among the groups of students. Each symbol represents one student, and lines represent the mean and standard deviation of each population. A Kruskal-Wallis test was used to assess differences among the populations, and p-values for comparisons among the groups in panels A, B, C, D, E, F, and G were 0.7506, 0.4714, 0.1795, and 0.1882, 0.5913, 0.0981, and 0.1602, respectively.
(TIF)

## Abbreviations

GRE: Graduate Record Examinations
GPA: Grade Point Average
ETS: Educational Testing Service
