# Supplementary figures and images for: Predictors of Student Productivity in Biomedical Graduate School Applications
Source: PLoS One. 2017 Jan 11;12(1):e0169121. doi: 10.1371/journal.pone.0169121 (PMC5226343; doi:10.1371/journal.pone.0169121)

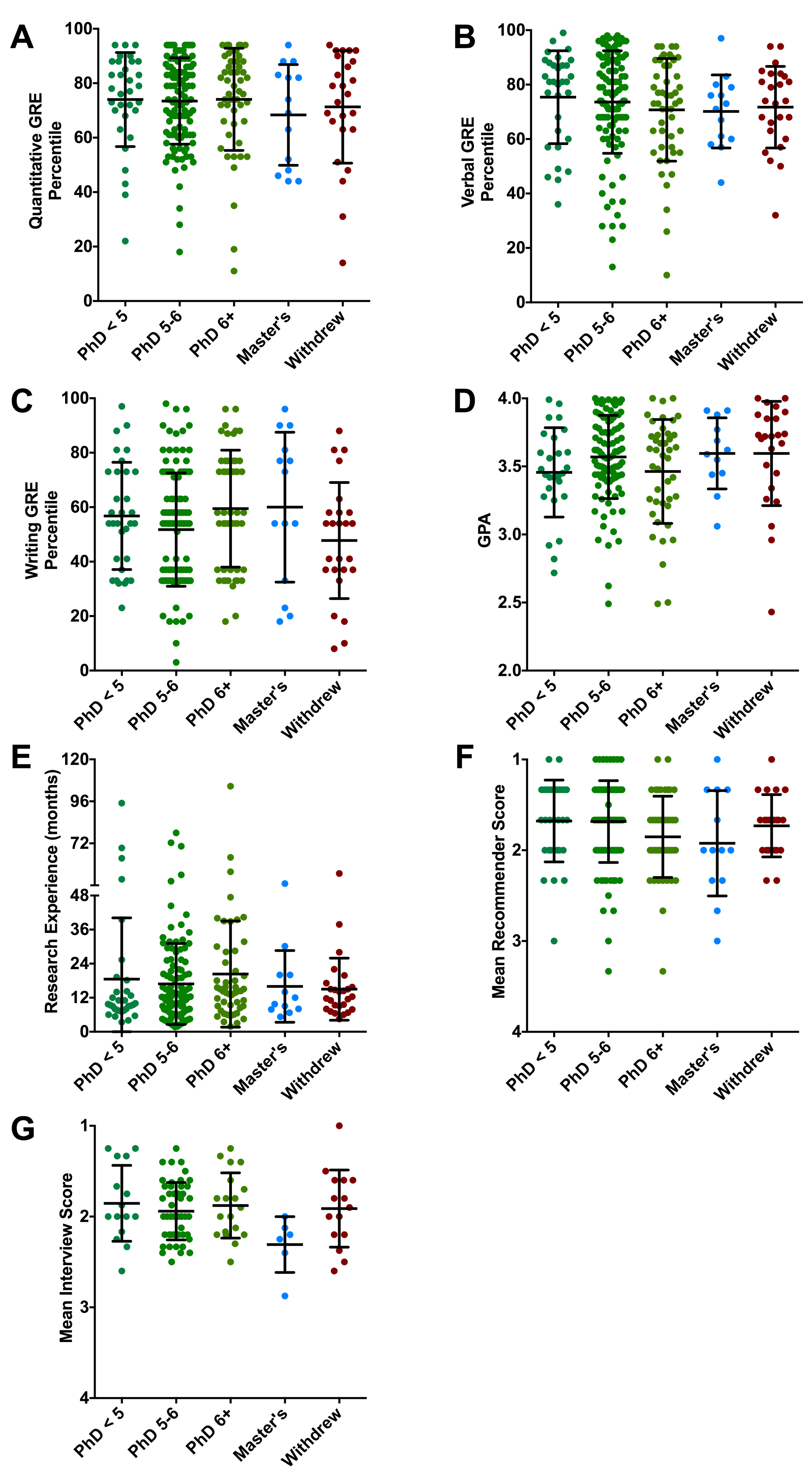

Supplement: S1 Fig — Students were assigned to groups based on their graduate school outcome, and students who completed their PhD were grouped based on time-to-degree (< 5 years, 4–5 years, > 5 years). (A) Quantitative GRE scores, (B) verbal GRE scores, (C) writing GRE scores, (D) GPA, (E) previous research experience, (F) recommender scores (1 = highest, 4 = lowest), and (G) one-on-one interview scores (1 = highest, 4 = lowest), were compared among the groups of students. Each symbol represents one student, and lines represent the mean and standard deviation of each population. A Kruskal-Wallis test was used to assess differences among the populations, and p-values for comparisons among the groups in panels A, B, C, D, E, F, and G were 0.7506, 0.4714, 0.1795, and 0.1882, 0.5913, 0.0981, and 0.1602, respectively. (TIF) [file pone.0169121.s001.tif]
